# Biosynthesis of Saxitoxin in Marine Dinoflagellates: An Omics Perspective

**DOI:** 10.3390/md18020103

**Published:** 2020-02-05

**Authors:** Muhamad Afiq Akbar, Nurul Yuziana Mohd Yusof, Noor Idayu Tahir, Asmat Ahmad, Gires Usup, Fathul Karim Sahrani, Hamidun Bunawan

**Affiliations:** 1 School of Bioscience and Biotechnology, Faculty of Science and Technology, Universiti Kebangsaan Malaysia, Bangi 43600, Malaysia; muhdafiq.akbar@gmail.com; 2 Department of Earth Science and Environment, Faculty of Science and Technology, Universiti Kebangsaan Malaysia, Bangi 43600, Malaysia; yuziana@ukm.edu.my (N.Y.M.Y.); fathul@ukm.edu.my (F.K.S.); 3 Malaysian Palm Oil Board, No 6, Persiaran Institusi, Bandar Baru Bangi, Kajang 43000, Selangor, Malaysia; idayu@mpob.gov.my; 4 University College Sabah Foundation, Jalan Sanzac, Kota Kinabalu 88100, Sabah, Malaysia; asmat@ukm.edu.my (A.A.); gires@ukm.edu.my (G.U.); 5 Institute of Systems Biology, Universiti Kebangsaan Malaysia, Bangi 43600, Malaysia

**Keywords:** saxitoxin, dinoflagellates, omics technologies, transcriptomics, proteomics, metabolomics, genomics

## Abstract

Saxitoxin is an alkaloid neurotoxin originally isolated from the clam *Saxidomus giganteus* in 1957. This group of neurotoxins is produced by several species of freshwater cyanobacteria and marine dinoflagellates. The saxitoxin biosynthesis pathway was described for the first time in the 1980s and, since then, it was studied in more than seven cyanobacterial genera, comprising 26 genes that form a cluster ranging from 25.7 kb to 35 kb in sequence length. Due to the complexity of the genomic landscape, saxitoxin biosynthesis in dinoflagellates remains unknown. In order to reveal and understand the dynamics of the activity in such impressive unicellular organisms with a complex genome, a strategy that can carefully engage them in a systems view is necessary. Advances in omics technology (the collective tools of biological sciences) facilitated high-throughput studies of the genome, transcriptome, proteome, and metabolome of dinoflagellates. The omics approach was utilized to address saxitoxin-producing dinoflagellates in response to environmental stresses to improve understanding of dinoflagellates gene–environment interactions. Therefore, in this review, the progress in understanding dinoflagellate saxitoxin biosynthesis using an omics approach is emphasized. Further potential applications of metabolomics and genomics to unravel novel insights into saxitoxin biosynthesis in dinoflagellates are also reviewed.

## 1. Introduction

Saxitoxin (STX) is a type of paralytic shellfish toxin (PST), and it is the most potent naturally occurring neurotoxic alkaloid known [1]. This compound is classified as a neurotoxin due to its ability to interact with voltage-gated sodium, potassium, and calcium channels, and it modulates the flux of these ions into various cell types [2]. In general, saxitoxin is produced by freshwater cyanobacteria and marine dinoflagellates [3]. Since the first discovery of saxitoxin in Alaska butter clams (*Saxidomus giganteus*) in 1957 [4], a total of 57 natural saxitoxin analogues, varying mainly in the substitution of the side-group moieties (R1–R5) of the perhydropurine tricyclic backbone, were reported, and those found in dinoflagellates are listed in Table 1. Based on the variation of the moieties, the saxitoxin can be classified as non-sulfated (neoSTX), mono-sulfated (GTX1-6), di-sulfated (C1-4), decarbamylated (dcSTX, dcneoSTX, dcGTXs1-4), and deoxy-decarbamoylated (doSTX, doGTXs1-3), causing different magnitudes of lethality [2,5]. Saxitoxin poisoning accounts for approximately 2000 cases worldwide annually with an average human mortality rate of 15% [3,6]. Most cases of human saxitoxin toxicosis are associated with the ingestion of contaminated seafood, e.g., bivalves which accumulate saxitoxins produced by marine dinoflagellates. Aside from general safety concerns, the financial impact of saxitoxin outbreaks is substantial. Pertinent organizations (for instance, shellfisheries, shoreline establishments, and other fish-related businesses) are heavily affected with evaluated yearly expense of United States dollars (USD) $895 million around the world utilized for monitoring and carrying out mitigation plans for saxitoxin-producing dinoflagellates [6]. 

**Figure 1:**
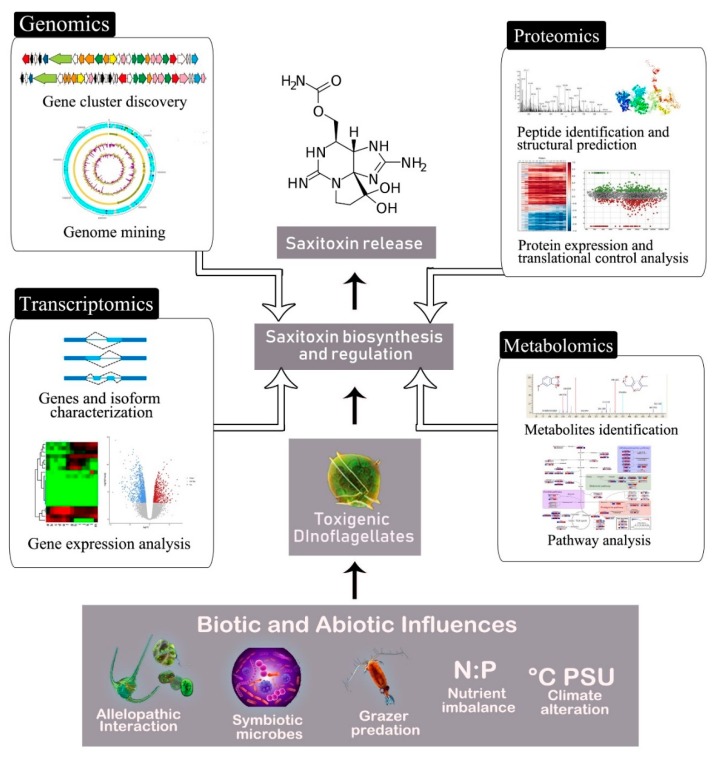
Decoding saxitoxin biosynthesis and its regulation via an omics approach. The production, release, and effect of saxitoxin by toxigenic dinoflagellates are influenced by their abiotic and biotic aquatic ecosystem components. This highly complex multi-organism and multi-stress environment within the context of saxitoxin synthesis can be grasped by the all-inclusive and high-throughput methods of omics.

Lately, studies of natural product biosynthesis were revolutionized by the unbiased and non-targeted universal detection, identification, and measurement of molecular fractions consisting of genes (genomics), messenger RNA (mRNA) (transcriptomics), proteins (proteomics), and metabolites (metabolomics), collectively dubbed as “omics” [23,24]. This is due to the capacity of omics technology and integrated methodologies to extract and appraise biomolecules at a global scale rather than targeting the individual genes, proteins, or metabolites via a traditional reductionist approach (Figure 1). A one-to-one interdisciplinary approach to linking the information element to its functional component was discussed by combining data obtained from one omics to another, e.g., transcriptomics and metabolomics in several microbial systems [25]. Integrated laboratory protocol allows the same sample to be utilized for several different omics analyses, creating an ideal platform for combined omics analysis to reveal co-regulation in biochemical networks [26]. The prospect of exploiting this strategy to unveil the complexity of saxitoxin biosynthesis by organisms of huge genomes (3–245 Gbp) such as dinoflagellates is promising. Hence, this review outlines and summarizes the recent progress of omics approach in understanding dinoflagellate saxitoxin molecular biosynthesis. Imperatives and challenges in adopting the methodology are also highlighted to allow its practical and impartial application for a sound and unequivocal biological interpretation.

## 2. Overview of Saxitoxin Molecular Biosynthesis and Gene Cluster

The first attempt to investigate the biosynthesis of saxitoxin was done by Shimizu et al. [27] using radioisotope tracing experiments. Their work suggested that saxitoxin is built from acetic acid, arginine, and *S*-adenosyl methionine (SAM), initiated by Claisen condensation of acetic acid and arginine. Later, the biosynthesis pathway for saxitoxin was revised and modified from the original proposed pathway based on the discovery and characterization of the saxitoxin gene cluster in saxitoxin-producing cyanobacteria via a genome walking approach and manual open reading frame (ORF) annotation [28]. The putative gene cluster for saxitoxin biosynthesis is encoded by 31 ORFs assigned to 26 proteins, stretching to 35 kb in length. This breakthrough was then followed by another finding of homologous gene clusters in several other cyanobacteria, ranging from 25.7 kb to 35 kb in length [29,30,31]. Based on the variation of gene clusters in saxitoxin-producing cyanobacteria, these genes were further classified into sets of core genes, regulator genes, tailoring genes, and transporter genes. 

**Figure 2:**
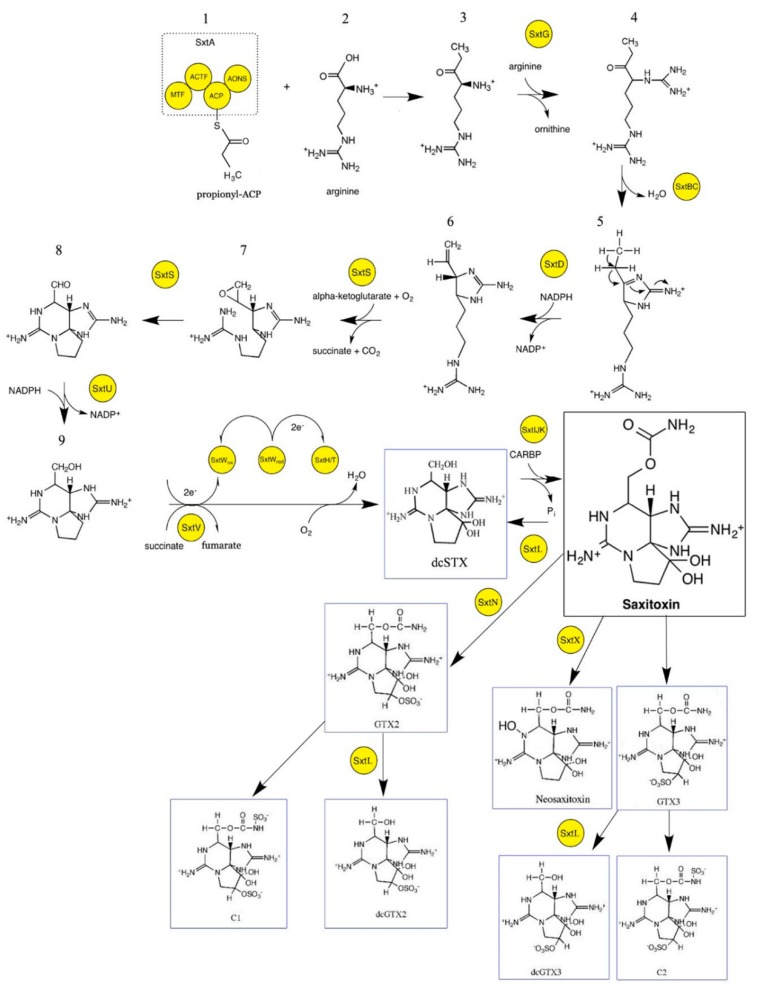
Putative pathway for saxitoxin biosynthesis in cyanobacteria. Proposed reactions are based on bioinformatics prediction incorporated from several studies [28,29,30]. A black box indicates a saxitoxin parent compound, and a blue box indicates selected saxitoxin analogues. Biosynthetic enzymes are highlighted in yellow circles.

In the proposed saxitoxin biosynthesis pathway (Figure 2), the biosynthesis begins by polyketide synthase (PKS)-like enzymes encoded by the gene *sxtA*, which contains four catalytic domains of SAM-dependent methyltransferase (*sxtA1*), GCN-5 related *N*-acetyltransferase (*sxtA2*), acyl carrier protein (*sxtA3*), and class II amidinotransferase (*sxtA4*). These PKS-like enzymes lack the trademark ketosynthase domain compared to the other typical PKSs [28]. *SxtA* initiates the biosynthesis via the following steps: (1) acetate from acetyl-coA is incorporated into an acyl carrier protein (ACP) which results in the formation of acetyl-ACP, (2) methylation of acetyl-ACP into propionyl-ACP by *sxtA1*, and (3) Claisen condensation of propionyl-ACP with arginine, which results in the formation of 4-amino-3-oxo-guanidinoheptane. This is followed by the transfer of the amidino group from arginine into 4-amino-3-oxo-guanidinoheptane by the amidinotransferase *sxtG*. This reaction results in the formation of 4,7-diguanidino-3-oxoheptane (intermediate 4). Subsequent conversion of intermediate 4 into intermediate 5 is aided by a cytidine deaminase-like enzymes encoded by *sxtB*. Sterol desaturase enzyme encoded by *sxtD* then likely introduces a double bond between C-1 and C-5 of intermediate 4, resulting in the 1,2-*H* shift between C-5 and C-6 (intermediate 5). Next, the *sxtS*, which has sequence similarity with nonheme iron 2-oxoglutarate-dependent dioxygenases, catalyzes the formation of intermediates 7 and 8 by performing successive epoxidation of the new double bond and the opening of the epoxide into an aldehyde with concomitant bicyclization. The subsequent reaction is executed by a short-chain alcohol dehydrogenase encoded by *sxtU* by reducing the terminal aldehyde group of intermediate 8, thus resulting in the formation of intermediate 9. The next reaction in saxitoxin biosynthesis involves the formation of the decarbamoylsaxitoxin (dcSTX) analogue by consecutive hydroxylation by phenylpropionate dioxygenase encoded by the gene *sxtH/T*. Oxygen reductase is required for the regeneration of these multicomponent enzymes of phenylpropionate dioxygenase after each catalytic reaction cycle. This requirement is fulfilled by a putative electron transport system encoded by the genes *sxtV* and *sxtW*. Biosynthesis of the parent molecule of saxitoxin is completed by the transfer of a carbamoyl group onto the free hydroxyl group of C-13 facilitated by a putative *O*-carbamoyltransferase encoded by the genes *sxtI*, *sxtJ*, and *sxtK*. Synthesis of other analogues from this saxitoxin parent molecule is aided by several tailoring enzymes. Different toxin profiles among species or strains of dinoflagellates are the outcome of the tailoring gene diversity. Analysis of several saxitoxin-producing cyanobacteria revealed the presence of several tailoring enzyme-encoding genes such as *sxtL*, *sxtN*, *sxtO*, and *sxtX*. Apart from these tailoring genes, a class of regulator genes is predicted to control the regulation of saxitoxin production in response to several environmental factors, as identified in the genome of *C. raciborskii* [28]. However, these genes were not found in other cyanobacterial genomes and, therefore, the saxitoxin regulator genes in the other cyanobacteria are thought to be located elsewhere in the genome, or their regulation may occur at the post-transcriptional level [3]. 

## 3. Recent Insight into Saxitoxin Biosynthesis through Transcriptomic Analysis 

The extraordinarily huge genome size of saxitoxin-producing dinoflagellates became a hindrance to genomics study, persuading researchers to turn to transcriptomics-based investigation as an alternative methodology that enables large-scale analysis of gene expression and transcriptome profiling [32,33]. Microarray and complementary DNA (cDNA) sequencing are the most common and preferred choices of transcriptomics approaches employed by researchers to investigate the saxitoxin biosynthesis in dinoflagellates. Since the identification of the saxitoxin gene cluster in cyanobacteria *C. raciborskii* T3 by Kellmann et al. [28], saxitoxin biosynthesis in dinoflagellates was inferred to follow the same pathway on the account of several intermediates identified in both cyanobacteria and dinoflagellates, suggesting the involvement of similar biosynthetic genes [34,35]. Since then, much effort was devoted to identifying candidate genes for saxitoxin biosynthesis in dinoflagellates using high-throughput transcriptome profiling, as well as a traditional PCR approach (Table 2). The earliest attempt to investigate the occurrence of these genes in dinoflagellates was undertaken by Yang et al. [36]. In silico search of *sxt* genes in the expressed sequence tag (EST) library of *Alexandrium minutum* that includes sequences from various physiological conditions and growth stages failed to identify any transcript with close homology to *sxt* genes. The inability to discover any homologous *sxt* genes in the *A. minutum* EST library is likely due to the limitation of available EST coverage [37]. 

Using an RNA-sequencing (RNA-seq) approach, several full-length *sxt* genes present in the transcripts of dinoflagellates *A. minutum* and *A. fundyense* were discovered [43]. A cDNA sequence analysis showed that these *sxt* genes in dinoflagellates differ from their cyanobacteria counterparts in terms of the presence of eukaryotic poly-A-tails, unique dinoflagellate spliced leader sequence, signal peptide, and GC content. These findings demonstrated that the *sxt* genes are indeed encoded in the genome of dinoflagellates and, thus, debunked the earlier belief that suggested the production of saxitoxin in dinoflagellates by intracellular Actinobacteria and Proteobacteria symbionts [6]. As the starting gene in saxitoxin biosynthesis, *sxtA* became a main target for study. Uniquely, two different transcript families of *sxtA* gene were identified in the *A. fundyense* transcriptome [43]. These two transcripts differed in their sequence length and encode different catalytic domains in which the longer transcript encodes all *sxtA* domains (*sxt*A1–*sxt*A4) as their cyanobacteria counterparts, while the shorter transcript encodes only domains *sxt*A1–*sxt*A3. Further assessment into the function of these genes led to the postulation that the long transcript of *sxtA4* might be directly involved in the biosynthesis of saxitoxin, as its occurrences were identified in many saxitoxin-producing dinoflagellates including *Gymnodinium catenatum* and *Pyrodinium bahamense* [42,43,48]. This is also in accordance with findings showing the correlation between gene *sxtA4* copy number in *A. minutum* and *A. ostenfeldii* genomes and their saxitoxin contents [50,51]. The reports from these transcriptomic studies enabled researchers to perform in situ detection of saxitoxin-producing dinoflagellates [43,52,53]. A more extensive survey by Hackett et al. [48] showed that the C-terminal region of *sxtA* is found exclusively in saxitoxin-producing dinoflagellates. A recent probe into various dinoflagellate species transcriptome library data from Marine Microbial Eukaryote Transcriptome Sequencing Program (MMETSP) also showed that the occurrence of domain *sxt*A4 is exclusive to saxitoxin-producing dinoflagellates including *G. catenatum* and *P. bahamense* and is highly conserved without paralogues, while the domain *sxt*A1 showed a widespread distribution in non-saxitoxin-producing dinoflagellates [54]. A more profound investigation utilizing phylogenetic approach revealed the presence of three paralogues for domain *sxt*A1 with one of the paralogues forming a highly corroborated clade of saxitoxin-producing dinoflagellates. The authors speculated that the *sxtA* originated from a common ancestor of *Alexandrium* spp., *G. catenatum*, and *P. bahamense* in the order of Peridiniales, 190 million years ago, based on similarity of the *sxtA4* domain in those species of these taxa [54,55]. However, the phylogeny of domain *sxtA1* does not appear to be consistent with the species phylogeny of these taxa, indicating that the origin of this gene is more complex, and there is the possibility of several events of gene duplication resulting in formation of several paralogues. The events of gene duplication may also occur from the recycling of mature mRNA back into the genomes, which is termed “retroposition”, resulting in the formation of retrogenes [56,57]. Retrogenes were widely studied for their involvement in dinoflagellate evolution during critical periods of extreme environmental changes, e.g., drastic temperature rise and fall, and they are associated with surprising characteristics, including their high persistence rate and their presence in large number in the genomes [56,58]. Nevertheless, the role of retroposition in shaping the evolution of saxitoxin and the formation of paralogues of domain *sxtA1* remains unexplored. In contrast to earlier findings, domain *sxtA4* was identified in several genomes of non-toxic *A. tamarense* group III and *Alexandrium australiense* (previously known as *A. tamarense* group V) [43,53,54]. However, to the best of our knowledge, there is no report on the expression of domain *sxtA4* from non-toxic dinoflagellates at the mRNA level. Zhang et al. [42] stated that the *sxtA4* domain is still in the genomes of non-toxic mutant *A. catanella*, although the expression of this gene was not detected in the RNA-seq data and q-PCR analysis, indicating that this gene is not transcribed into mRNA. Murray et al. [54] also found out that domain *sxtA4* is not detected in the transcriptome library of *A. tamarense* group III despite the presence of this domain in its genome [43]. This rather contradictory result might be due to the silencing of the saxitoxin biosynthesis gene cluster expression in non-saxitoxin-producing dinoflagellates. Several reports on the presence of silent biosynthetic gene clusters recently surfaced, including in several marine microorganism, although no report is yet found for dinoflagellates [59]. There is also another possibility of additional gene existence apart from the *sxtA4* domain, which are crucial for the biosynthesis of saxitoxin and which are absent in these non-toxic strains. 

Recognizing that the putative genes involved in saxitoxin biosynthesis possess various homologues in other diverse classes of organisms, Hackett et al. [48] suggested that the combination of enzymatic reactions encoded by these genes in a particular arrangement would result in saxitoxin production in dinoflagellates. This hypothesis is also supported by a later transcriptome analysis of non-saxitoxin-producing *Scrippsiella trochoidea* dinoflagellate that documented 113 transcripts perceived as homologues of *sxt* genes, covering 17 of the 34 genes found in *C. raciborskii sxt* genes, including the short isoform of *sxt*A1–A3 [60]. Thus, the homologue of *sxt* genes in dinoflagellates except for domain *sxtA4* is not exclusive to toxin-producing species, and we hypothesize that these genes may function in regulating the cellular metabolism or synthesizing other secondary metabolites. For example, *sxtI* which encodes the protein *O*-carbamoyltransferase is known to be involved in the biosynthesis of other secondary metabolites such as antibiotics [61]. Since the identification of a complete saxitoxin biosynthesis gene cluster is still not accomplished in dinoflagellates, the role of *sxtA4* domains in some non-toxic strains cannot be further explained. 

*SxtA* and *sxtG* are both characterized as core genes for the biosynthesis of saxitoxin in dinoflagellates, and they were studied extensively compared to other *sxt* genes (Table 2). The structure of several *sxtG* mRNA transcripts was firstly described in detail by Orr et al. [44] with a structure similar to a previous *sxtA* gene, including their monocistronic mRNA, typical eukaryotic poly(A) tails, and unique dinoflagellate spliced leader sequence, all encoded in a nuclear genome. The *sxtG* genomic sequence contains a noncanonical intron that showed high interspecies and low intraspecies variability [44]. Hackett et al. [48] and Murray et al. [54] demonstrated that occurrences of *sxtG* are unique to dinoflagellates producing saxitoxin, including *G. catenatum* and *P. bahamense*, and it was not detected in any non-saxitoxin-producing dinoflagellates including those from the genus *Alexandrium*. However, the expression of *sxtG* mRNA was detected in three non-saxitoxin-producing *Alexandrium* species, although no such gene was reported in dinoflagellates other than *Alexandrium* spp. and *G. catenatum* [44]. As no stop codon was observed in the sequence and a low genomic copy was amplified from its genome, the mRNA of *sxtG* from non-saxitoxin-producing *Alexandrium* spp. detected in this study may not be functional. The possible interference from the use of different transcriptome profiling technologies and inconsistent harvesting times for each of the study cannot be ruled out. A more systematic and wider coverage of sequencing might be needed to address these rather varying results. Phylogeny analysis of dinoflagellate *sxtG* genes formed a highly conserved and fully supported clade, separating the genus from other dinoflagellates and bacteria with amidinotransferase enzymes [44,54]. Selection analysis of *sxtG* and other amidinotransferase branching also revealed that the *sxtG* branch is under negative selection, whereas other phylogeny branches including dinoflagellate amidinotransferase clades showed a significant positive selection [54]. A similar pattern of natural selection also was observed in the *sxtA1* clade that is made of saxitoxin-producing dinoflagellates [54].

Out of 14 “core” genes for saxitoxin biosynthesis characterized from cyanobacteria, only nine of these essential genes were putatively identified within saxitoxin-producing dinoflagellates with a certain degree of similarity (Table 2). Apart from *sxtA* and *sxtG*, the role of these genes in the biosynthesis of dinoflagellates saxitoxin remains poorly understood. These genes were putatively identified based on the closest BLAST hits, and none of them were extensively described for their domain architecture and sequence analysis. These *sxt* genes (other than *sxtA* and *sxtG*) might have different functions in dinoflagellates, as these genes align at lower significant levels to *sxt* genes from cyanobacteria [43]. Phylogenetic analysis in *A. tamarense* demonstrated that these three genes (*sxtA*, *sxtG*, and *sxtB*) are closely related to cyanobacteria and catalyze in the first three steps of the saxitoxin biosynthesis [48]. Other dinoflagellate *sxt* genes (*sxtD*, *sxtS*, *sxtU*, *sxtH/T*, and *sxtI*) showed poor phylogenetic relationship with *sxt* genes from cyanobacteria, and these complicate understanding the origin of these genes [48]. Dinoflagellates might have a different set of genes for saxitoxin biosynthesis, and their evolution is independent from cyanobacteria. The current hypothesis on the origin of saxitoxin biosynthesis in dinoflagellates based on *sxtA* and *sxtG* suggests that *sxt* genes were acquired independently in both dinoflagellates and cyanobacteria [6,44,48]. Considering collective results from Orr et al. [44] and Stuken et al. [43], *sxt* genes in dinoflagellates were acquired via horizontal gene transfer from Actinobacteria and Proteobacteria. Various aspects of the evolutionary relationship of dinoflagellates saxitoxin biosynthesis genes, including sources of these genes, phylogenetic inference, and events of horizontal gene transfer, were extensively reviewed by Orr et al. [6].

In addition to the identification of toxin synthesis genes based on the cyanobacteria gene cluster, comparative transcriptome analysis became a valuable tool for dissecting the role of differently expressed genes (DEGs) among similar biological samples under different biological conditions in order to obtain a wider perspective of saxitoxin gene discovery in dinoflagellates (Table 3). The differentially expressed genes between the toxin and non-toxin dinoflagellates strains can also provide useful insight into the expression of saxitoxin genes. Comparative transcriptome analysis using microarrays on toxic and non-toxic strains of *A. minutum* revealed novel candidate genes for saxitoxin biosynthesis [36]. However, due to the lack of a homology sequence for dinoflagellates, most of these genes remain unannotated. A more recent comparative transcriptome analysis using high-throughput RNA-sequencing of saxitoxin producing *A. catenella* and its non-toxic mutant revealed 35 differently expressed genes among these strains [42]. Among the DEGs, *sxtA4* genes were downregulated in non-toxic mutants, further supporting the hypothesis for involvement of this gene in saxitoxin biosynthesis. Apart from that, other DEGs were found to be mainly associated with photosynthesis, carbon fixation, and amino-acid metabolism. 

Intracellular toxin content in dinoflagellates is closely linked to nutritional status [62,63]. With this information in view, the microarray gene expression study found that most of the differently expressed genes are involved in the dinoflagellate core metabolic process with no immediate connection toward saxitoxin biosynthesis [62]. Nonetheless, two of the non-annotated genes showed a consistence regulation pattern with cellular saxitoxin content. Despite the numerous studies connecting nutritional status, for example, nitrogen and phosphorus with respect to cell saxitoxin content, this relationship is poorly portrayed with transcriptomics studies from saxitoxin-producing dinoflagellates of *A. minutum*, *A. tamarense*, and *A. fundyense* [62,64,65]. Altogether, these studies were unable to find the correlation between dinoflagellate core metabolism and its impact on saxitoxin biosynthesis. However, it is noteworthy that none of these studies utilized the recent next-generation RNA-sequencing approach with deeper transcriptome coverage, which may provide progressively significant outcome compared to previous techniques. 

The degree of dependency to which dinoflagellates alter the saxitoxin production level under transcriptional responses is yet to be resolved. For example, the gene expression pattern for *sxtA4* in *A. catenella* examined by RT-qPCR did not significantly change under different growth stages, even though the saxitoxin production was significantly altered [45]. A similar expression pattern was also observed in *sxtA* and *sxtG* genes in *A. minutum* [49]. Examination by RNA-seq on the *A. catenella* transcriptome also revealed that most of the putative toxin gene transcripts varied insignificantly at different toxin biosynthesis stages [46]. All these results might suggest that saxitoxin production is ultimately regulated at a translational or post-translational level. Only a small portion of dinoflagellates genes are now presumed to be regulated at the transcriptome level [46]. For instance, under nitrogen starvation, only 220 genes were found to be differentially expressed in the dinoflagellate *Amphidinium carterae* [66]. Similarly, RNA-sequencing of the dinoflagellate *S. trochoidea* revealed only 178 transcripts from a total of 107,415 contigs that were differentially expressed under nitrogen stress [60]. Lin [67] suggested that limited transcriptional regulation in dinoflagellates is likely due to the lack of histone proteins which are thought to regulate gene expression in eukaryotes. Several recent studies illustrated that histone proteins may regulate gene expression for the production of secondary metabolites in some fungi [68,69]. Even though genes that encode histone proteins were identified in the dinoflagellate transcriptome, including a full suite of histone proteins, these genes might be expressed at a lower level and may play a limited role in regulating the gene expression [70].

## 4. Translational Control in Dinoflagellates and Its Implication on Saxitoxin Biosynthesis

As previously mentioned, the changes in dinoflagellate gene expression were widely studied via several methods of transcriptomics approaches, such as microarrays and high-throughput RNA-sequencing. Limited transcriptional regulation in the dinoflagellates indicated that saxitoxin biosynthesis might be regulated at the translational level. Moreover, the discovery of a unique spliced leader sequence in dinoflagellate transcripts [72,73] suggested that most dinoflagellate genes are regulated at the post-transcriptional level as in some eukaryotes using a trans-splicing mechanism [74,75]. Regulation of saxitoxin biosynthesis at the post-transcriptional level via trans-splicing is additionally bolstered by sequence analysis of *sxtA4* and *sxtG* transcripts that demonstrated the presence of a dinoflagellate spliced leader sequence in these genes [43,44]. Additionally, apart from the coding RNA transcripts in dinoflagellates, there are many non-coding RNA transcripts in dinoflagellates, e.g., microRNA (miRNA) [76,77,78,79,80]. These miRNAs were observed to post-transcriptionally direct the expression of several genes associated with different biological processes in dinoflagellates [79,80]. Given their potential role in gene regulation, the significance of miRNAs in regulating saxitoxin biosynthesis cannot be ignored, as several miRNAs were documented in saxitoxin-producing dinoflagellates [77,78]. In most eukaryotes, the protein translational mechanism begins with eukaryotic translation initiation factor 4E (eIF4E) through its interaction with the 5’-cap structure of mRNA [81]. Abundance and activities of protein elF4E often become the target for translational control, as seen in the phosphorylation of protein elF4E that regulates the circadian protein expression [82]. Recent discovery of an extensive transcript encoding the protein elF4E family in dinoflagellates provides additional evidence for substantial translational control in dinoflagellates [83]. 

More reports for translational control in dinoflagellates were found (Table 4), leading researchers to conclude that the expression of *sxt* genes in dinoflagellates is controlled at the post-transcriptional or post-translational level owing to the inconsistent expression level at the transcript level documented by previous studies [46,49] and the presence of a dinoflagellate splice-leader sequence in at least two core genes for saxitoxin biosynthesis [43,44]. However, as we are still traversing in the unknown genetic territory, gene expression at the transcript level in dinoflagellates cannot be completely overlooked. Although limited, several studies documented effective regulation of dinoflagellate genes at the transcript level [32].

## 5. Proteomics Insight into Saxitoxin Biosynthesis

Compared to RNA levels, protein abundance measurements are believed to be more closely correlated to catalytic capability and phenotypic observation of a cell [88]. The proteomics approach also enables researchers to discover biomarkers indicative of cell status, including toxicity. Thus, the study of proteins appears to be promising in facilitating the establishment of a molecular mechanism behind the regulation and biosynthesis of saxitoxin in dinoflagellates at the translational level. The technology and informatics advancement of the omics age enables researchers to examine hundreds to thousands of proteins simultaneously either via traditional 2-DE gel-based approach (two-dimensional gel electrophoresis), 2D DIGE (difference gel electrophoresis), label-free high-throughput shotgun liquid chromatography–tandem mass spectrometry (LC–MS/MS), or chemically labeled LC–MS/MS such as tandem mass tags (TMT) or isobaric tags for relative and absolute quantification (iTRAQ) [3,89,90]. 

**Figure 3:**
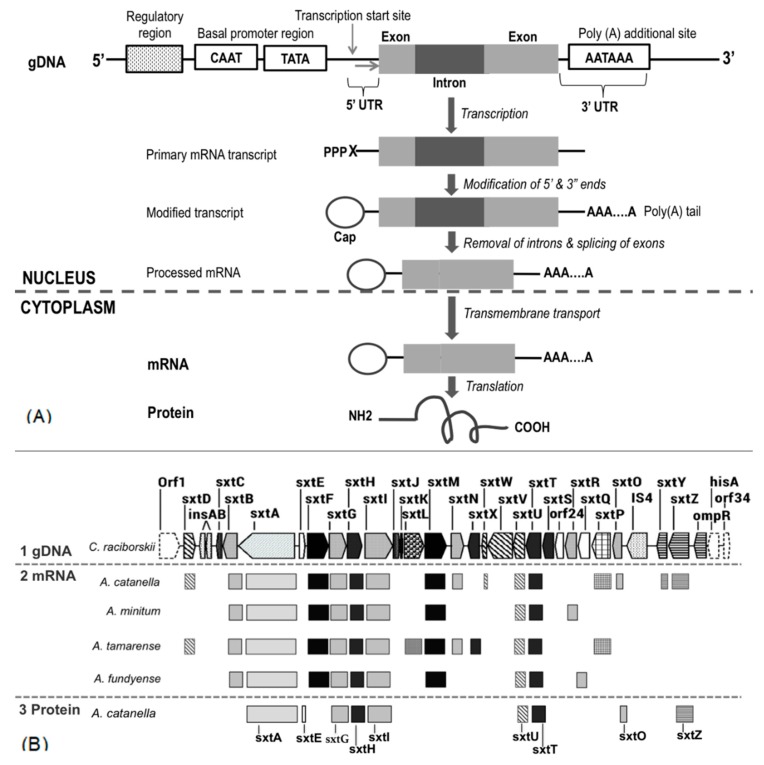
(**A**) Central dogma of molecular biology describing the flow of genetic information from a double-stranded genomic DNA template to post-translationally modified proteins. In the nucleus, the double-stranded DNA template is transcribed into a single-stranded pre-messenger RNA (mRNA), which is further processed through steps of modification of the 5’ and 3’ ends, polyadenylation, removal of introns, and splicing of exons. The mature mRNA is exported to the cytoplasm for translation to an amino-acid sequence, which is folded and/or post-translationally modified and subcellularly localized as a functional protein. (**B**) Information of *sxt* molecules at the level of genomic DNA (gDNA), mRNA and protein. To date, the *sxt* gene cluster was successfully identified only in cyanobacteria species [28]. However, through transcriptomic analysis, several numbers of expressed mRNA were detected from several dinoflagellate species, as described in Table 2. Based on proteomic analysis, nine proteins encoded by *sxt* genes of *A. catanella* were identified as described in Section 5.

Early studies on saxitoxin-producing dinoflagellates proteome focused on identifying candidate proteins that may function as biomarkers for its toxicity [91]. Combination of computational analysis on 2-DE gel combined with MALDI-TOF MS detection and N-terminal amino-acid sequencing revealed a candidate protein of T1, which showed a high correlation with toxicity in several strains of *A. tamarense* and *A. minutum* [91,92]. However, the role of this protein was unascertained, and further functional analysis is needed to determine the role of T1 protein in the saxitoxin biosynthesis. In another finding, polyketide synthase, histidine kinase, and chaperon-like proteins encoded by *sxtA*, *sxtZ*, and *sxtE*, respectively, were downregulated in the non-toxic strain of *A. catenella*, indicating the direct involvement of these proteins in saxitoxin biosynthesis. The upregulation of dihydrolipoamide succinyltransferase protein and succinyl-CoA synthetase subunit beta might reduce the flow of succinate, which is an important precursor for saxitoxin biosynthesis, thus resulting in lower toxin production [93]. Furthermore, nine putative proteins in saxitoxin biosynthesis (methionine *S*-adenosyltransferase, chloroplast ferredoxin-NADP^+^ reductase, *S*-adenosyl homocysteinase, adenosylhomocysteinase, ornithine carbamoyltransferase, inorganic pyrophosphatase, sulfotransferase, alcohol dehydrogenase, and arginine deiminase) were identified in the proteome of saxitoxin-producing *A. catenella*, and their expression patterns throughout the different toxin biosynthesis stages showed a complex interaction network, proposing that these proteins might contribute to saxitoxin biosynthesis in a mutually promotive way [94] (Figure 3). Together, these results further suggest that saxitoxin biosynthesis in dinoflagellates is regulated at the translational level.

Differentially expressed protein analysis from saxitoxin-producing *A. catenella* and its non-toxic mutant, which lost the ability to form toxin during routine culture maintenance, showed suppression of several biological processes of amino-acid biosynthesis, fatty-acid biosynthesis, carbohydrate metabolism, photosynthesis, and bioluminescence, as well as their enhancement in the non-toxic mutant proteome [93,95]. These studies outlined that the elevated photosynthesis rate in the non-toxic mutant of *A. catenella* might contribute to its higher growth rate compared to its toxic counterpart. In another investigation, the proteomics analysis of mutated saxitoxin-producing *A. catenella* exposed to a cell-cycle inhibitor colchicine revealed the upregulation of several proteins involved in photosynthesis and carotenoid biosynthesis during the arrested toxin production stage, indicating the channeling of nitrogen compounds originally allocated for saxitoxin biosynthesis toward the synthesis of carotenoid and nitrogenous chlorophyll [96]. However, these claims can be contended with a finding by Jiang et al. [97] of several photosynthesis-related proteins having enhanced expression during the highly toxic period of saxitoxin-producing *A. tamarense*, which can be reflected back to a study by Etheridge and Roesler [98] that was unable to find a significant correlation between photosynthesis rate and toxin production in the toxic *A. fundyense*. Thus, it is unclear whether the rate of photosynthesis and growth rate directly affect saxitoxin biosynthesis in dinoflagellates or if the observations were just the effects of random mutation as shown in the case of the non-toxic mutant of *A. catenella*. The loss of toxicity in the *A. lusitanicum* strain even seems to be associated with a reduced growth rate, while comparable growth rates were observed between saxitoxin-producing *A. tamarense* and its non-toxic mutant [99,100]. Since the exact mutation localization in the genomic sequence of these strains is yet to be determined, and the fitness cost and tradeoffs between toxin biosynthesis and cell development of dinoflagellates are inadequately understood, differentially expressed proteins between these strains must be interpreted carefully. 

## 6. Metabolomics within the Context of Saxitoxin Biosynthesis: An Unexplored Approach? 

Our present comprehension is yet to pinpoint the definite cause of these adjustments in saxitoxin biosynthesis, i.e., whether it is activated by changes in cellular metabolism, a consequence of pleiotropic alteration, or adaptation in the ecological condition. It is postulated that these changes may exert some effect on the central metabolism of dinoflagellates that then stimulates important precursor(s) for secondary metabolite biosynthesis as observed in other organisms, for example, plants, fungi, and marine diatoms [101,102,103]. In the hopes of linking the genotype to phenotype, metabolomics offers several advantages of unbiased snapshots of biochemical status, i.e., a reflection of the happenings at a functional level [104,105]. While metabolism was discussed since more than 700 years ago, “metabolomics” was first coined in 2001 and is considered the most recent component of the omics field, focusing on a snapshot measurement of small molecules or metabolites (<1500 Da) in a biological system under a given set of conditions [106,107,108]. The status of a metabolome as the ultimate end product of cellular regulatory processes gives researchers the opportunity to detect changes that arise from physiological or environmental events over shorter time scales, in view of the fact that the level of metabolite changes are believed to be highly correlated with organism phenotype [105,109]. In this sense, it is also important to note that metabolomics is more sensitive to time than the other omics [110]. The constantly evolving and yet subtle changes in a living system are measured by metabolomics, an approach that is appropriately viewed as “genetic information in motion” [111]. The utilization of metabolomics as a discovery platform permitted the elucidation of the polyketide-like synthase and the resulting α-amino ketones the saxitoxin biosynthesis [112]. Another targeted assessment of the saxitoxin biosynthetic pathway was conducted using a nitrogen isotope tracer of sodium nitrate as a substrate to investigate nitrogen incorporation into the precursor, the biosynthetic intermediates, and the saxitoxin analogues in both toxin- and non-toxin-producing dinoflagellate species of *A. catenella, Alexandrium insuetum, Prorocentrum triestinum*, and *G. catenatum* [113]. The study supported the proposed biosynthetic pathway in Figure 2, and the quantitation of labeled and non-labeled arginine, saxitoxin biosynthetic intermediates, and the main saxitoxin analogues revealed the pool size of the amino acids and important metabolites that could provide information on the variability of toxin production under varying nutrient status.

It is anticipated that numerous exogenous signaling metabolites with plausible influence on saxitoxin production in dinoflagellates are to be detected and measured by means of metabolomics [114,115]. There are currently a limited number of reports that can be found on the external chemical cues for saxitoxin production in dinoflagellates. *A. minutum* boosted a multifold of its saxitoxin production, ensuing the prompts from copepodamides released by predatory zooplankton *Centropages typicus*, even though the zooplankton itself is resistant to saxitoxin toxicity [116]. These copepodamides are polar lipids which were also found to trigger higher poison output in domoic acid-producing diatoms as a defense mechanism against the herbivorous copepods [117]. The alteration or substitution of local microbial community was demonstrated to cause noteworthy changes in saxitoxin production by the athecate dinoflagellate *G. catenatum* [118]. This outcome is in agreement with the discovery of a later investigation, in which a few groups of bacteria were found to be present consistently among several strains of *A. ostenfeldii, A. minutum*, and *A. tamarense* [119]. The fascinating coexistence of dinoflagellates and bacteria in their phycosphere even led to the formulation of theories of saxitoxin biosynthesis by symbiotic bacteria living within the dinoflagellates. However, this notion was rejected when the structure of *sxt* gene transcripts was represented, avowing that these genes are encoded in the genomes of dinoflagellates, and symbiont microbes may possibly assume an external chore in regulating saxitoxin production [43,44]. The precise physiological and biochemical impacts of these bacteria on dinoflagellates, as well as their ability to produce any signaling metabolites as a part of complex nutrient or deterrent exchange to affect the regulation of saxitoxin production, warrant a thorough analysis as attempted by Wang et al. [120] with the co-culture set-up for *Dinoroseobacter shibae* and *Prorocentrum minimum* but conjoined with metabolomics. Alongside the lethal saxitoxins of purine alkaloids, dinoflagellates also produce other toxins of spirolides, macrolides, and cyclic polyethers, for which definitive roles are still incognito to researchers. The arduous molecular characterization of their biosynthetic pathways can only be effectively uncovered with biochemical information [121].

## 7. Future Directions and Conclusions

The examination of dinoflagellate genomes is limited to known genes identified utilizing random approaches [122,123,124,125] with the exception of several high-quality draft genomes from the genus *Symbiodinium* [79,126,127]. While these works contributed important information in many aspects, the large and complex genome structure of dinoflagellates, especially in the view of the molecular mechanism for saxitoxin biosynthesis, is still uncracked. With the high gene copy number genome characteristic, copy number variation (CNV) emerged as another noteworthy source for phenotypic diversity within organism populations, as displayed in alveolate *Plasmodium falciparum* populations which share several similar genomic architectures with dinoflagellates [128]. Considered significant for adaptive evolution, CNV between dinoflagellate saxitoxin genes can be more confidently asserted together with its role, whether for adaptation or other responses, with the availability of genome sequence. In saxitoxin-producing cyanobacteria, the genes encoding saxitoxin biosynthesis are adjacent to each other in their genome as a biosynthetic gene cluster (BGC) [28,30]. However, *sxt* genes in dinoflagellates may or may not occur in a cluster. To date, several gene clusters were described in dinoflagellates including small nuclear RNA (snRNA), a hybrid NRPS–PKS, and mycosporine-like amino acid (MAA) gene clusters [126,129,130]. With the advancement of third-generation sequencing, such as single-molecule real-time (SMRT) sequencing by Pacific Biosystems (PacBio) and nanopore sequencing by Oxford Nanopore Techniques (ONT), challenges in sequencing large genomes to some extent will be overcome [131,132]. By utilizing long reads, third-generation sequencing lessens the burden for large genome assembly, allowing significant improvements of genome assemblies in repetitive regions via an overlap–layout–consensus approach [133]. We believe that the whole-genome sequence of saxitoxin-producing dinoflagellates is on the horizon, allowing the prediction of all genes, as well as microRNA and non-coding RNA, and revealing the architecture of the genome arrangement and its genomic landscape of many other genetic features.

Saxitoxin biosynthesis regulation is indeed a complex process, controlled by several environmental cues that are yet to be defined. At this point in time, omics-based studies in dinoflagellates focusing on saxitoxin biosynthesis are still sporadic and far from coming to a concrete closure. As the aquatic ecosystem is a continuum, the occurrence of saxitoxin-producing species will continue to expand geographically. This overwhelming situation combined with an unexplored genome might well be tackled with a bona fide integrated omics approach, in which the specimens are prepared and analyzed cohesively up to the data interpretation. Instead of combining layers of information from each of the omics carried out separately, integrated protocols of biomolecule extraction allow better correlation and cost efficiency from expending the same starting material [134]. Data analysis from this same set of specimens can then be analyzed in an algorithm, e.g., multivariate correlation, network analysis, and Granger causality, which allows many-to-many association, thereby interpreting the omics as a whole entity [135,136]. The ongoing development of these cutting-edge omics technologies and improvements in omics data mining will likely expand the omics-based studies in dinoflagellates in the near future, especially in decoding the saxitoxin biosynthesis enigma.

## Figures and Tables

**Table 1:** Structure of saxitoxin (STX) and side-group moieties produced by marine dinoflagellates. Modified and adapted from Wiese et al. [1].

Structure of Saxitoxin (STX)							
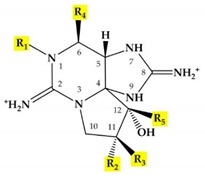							
Analogues	R_1_	R_2_	R_3_	R_4_	R_5_	Sources	Reference
STX	H	H	H	OCONH_2_	OH	*Alexandrium andersoni, Alexandrium catenella, Alexandrium fundyense, Alexandrium tamarense, Gymnodinium catenatum, Pyrodinium bahamense*	[7,8,9,10,11,12]
neoSTX	OH	H	H	OCONH_2_	OH	*A. andersoni, A. catenella, A. fundyense, A. tamarense, G. catenatum, P. bahamense*	[7,8,9,10,11,12]
**Mono-sulfated**							
GTX1	OH	H	OSO_3_^−^	OCONH_2_	OH	*A. catenella, A. fundyense, A. minutum, A. tamarense, G. catenatum*	[8,9,10,12,13]
GTX2	H	H	OSO_3_^−^	OCONH_2_	OH	*A. catenella, A. fundyense, A. minutum, Alexandrium ostenfeldii, A. tamarense, G. catenatum*	[8,10,12,13,14,15]
GTX3	H	OSO_3_^−^	H	OCONH_2_	OH	*A. catenella, A. fundyense, A. minutum, A. ostenfeldii, A. tamarense, G. catenatum*	[8,9,12,13,14,15]
GTX4	OH	OSO_3_^−^	H	OCONH_2_	OH	*A. catenella, A. fundyense, A. minutum, A. tamarense, G. catenatum*	[8,9,10,13,16]
GTX5 (B1)	H	H	H	OCONHSO_3_^−^	OH	*A. catenella, A. fundyense, A. tamarense, G. catenatum, P. bahamense*	[8,9,10,11,12]
GTX6 (B2)	OH	H	H	OCONHSO_3_^−^	OH	*A. catenella, A. fundyense, A. ostenfeldii, A. tamarense, G. catenatum, P. bahamense*	[8,9,10,11,12]
**Di-sulfated**							
C1	H	H	OSO_3_^−^	OCONHSO_3_^−^	OH	*A. catenella, A. fundyense, A. ostenfeldii, A. tamarense, G. catenatum*	[8,9,10,12,15,17,18]
C2	H	OSO_3_^−^	H	OCONHSO_3_^−^	OH	*A. catenella, A. fundyense, A. ostenfeldii, A. tamarense, G. catenatum*	[8,9,10,12,15,18]
C3	OH	H	OSO_3_^−^	OCONHSO_3_^−^	OH	*A. catenella, G. catenatum*	[14,19]
C4	OH	OSO_3_^−^	H	OCONHSO_3_^−^	OH	*A. catenella, G. catenatum*	[14,19]
**Decarbamoylated**							
dcSTX	H	H	H	OH	OH	*A. catenella, G. catenatum, P. bahamense*	[8,11,12]
dcneoSTX	OH	H	H	OH	OH	*A. tamarense*	[20]
dcGTX1	OH	H	OSO_3_^−^	OH	OH	*G. catenatum*	[21]
dcGTX2	H	H	OSO_3_^−^	OH	OH	*A. catenella, A. fundyense, G. catenatum*	[8,12,17]
dcGTX3	H	OSO_3_^−^	H	OH	OH	*A. catenella, A. fundyense, G. catenatum*	[8,12,17]
dcGTX4	OH	OSO_3_^−^	H	OH	OH	*G. catenatum*	[21]
**Deoxy-decarbamoylated**							
doSTX	H	H	H	H	OH	*G. catenatum*	[22]
doGTX1	OH	H	OSO_3_^−^	H	OH	*G. catenatum*	[22]
doGTX2	H	H	OSO_3_^−^	H	OH	*G. catenatum*	[22]
**Mono-hydroxybenzoate Analogues**							
GC1	H	H	OSO_3_^−^	OCOPhOH	OH	*G. catenatum*	[21]
GC2	H	OSO_3_^−^	H	OCOPhOH	OH	*G. catenatum*	[21]
GC3	H	H	H	OCOPhOH	OH	*G. catenatum*	[21]
^*^ GC4	OH	H	OSO_3_^−^	OCOPhOH	OH	*G. catenatum*	[19]
^*^ GC5	OH	OSO_3_^−^	H	OCOPhOH	OH	*G. catenatum*	[19]
^*^ GC6	OH	H	H	OCOPhOH	OH	*G. catenatum*	[19]
**Di-hydroxybenzoate Analogues**							
^+^ GC1a	H	H	OSO_3_^−^	DHB	OH	*G. catenatum*	[19]
^+^ GC2a	H	OSO_3_^−^	H	DHB	OH	*G. catenatum*	[19]
^+^ GC3a	H	H	H	DHB	OH	*G. catenatum*	[19]
^+^ GC4a	OH	H	OSO_3_^−^	DHB	OH	*G. catenatum*	[19]
^+^ GC5a	OH	OSO_3_^−^	H	DHB	OH	*G. catenatum*	[19]
^+^ GC6a	OH	H	H	DHB	OH	*G. catenatum*	[19]
**Sulfated Benzoate Analogues**							
^+^ GC1b	H	H	OSO_3_^−^	SB	OH	*G. catenatum*	[19]
^+^ GC2b	H	OSO_3_^−^	H	SB	OH	*G. catenatum*	[19]
^+^ GC3b	H	H	H	SB	OH	*G. catenatum*	[19]
^+^ GC4b	OH	H	OSO_3_^−^	SB	OH	*G. catenatum*	[19]
^+^ GC5b	OH	OSO_3_^−^	H	SB	OH	*G. catenatum*	[19]
^+^ GC6b	OH	H	H	SB	OH	*G. catenatum*	[19]

* Not structurally characterized. ^+^ R_4_ group putatively assigned based on major ions obtained via mass spectrometry analysis.

**Table 2:** List of candidate *sxt* genes based on the cyanobacteria saxitoxin gene cluster and their occurrence in saxitoxin-producing dinoflagellates putatively identified through high-throughput transcriptome profiling and PCR. ACP—acyl carrier protein; PST—paralytic shellfish toxin.

Role	Genes	Size (bp)	Putative Function	Species	Reference
Core genes	*sx* *tA*	3702–3735	Methylation, loading of ACP, Claisen condensation	*A. minutum* (AIN34673.1)*, A. catanella* (AIR95660.1)*, A. tamarense* (AIL29903.1)*, A. fundyense* (ADY62525.1)*, A. ostenfeldii, Alexandrium tamiyavanichii, G. catenatum* (AVV62437.1)*, P. bahamense* (QEX95300.1)	[38,39,40,41,42,43,44,45,46,47,48,49]
	*sxtB*	954–975	Cyclization	*A. catenella, A. fundyense, A. minutum, A. tamarense*	[42,43,46,48]
	*sxtC*	282–351	Regulatory	-	-
	*sxtD*	756–798	Desaturation	*A. catenella, A. tamarense*	[42,46,48]
	*sxtG*	1131	Amidinotransfer	*A. minutum* (AGC84341.1)*, A. catenella* (AGC84338.1)*, A. fundyense* (AGC84339.1)*, A. tamarense* (AGC84356.1)*, G. catenatum* (AGC84343.1)*, P. bahamense* (JAG92740.1)	[40,42,43,44,46,48,49]
	*sxtH/T*	1002–1059	C-12 hydroxylation	*A. catenella, A. fundyense, A. minutum, A. tamarense*	[42,43,46,48]
	*sxtI*	1836–1923	Carbamoylation	*A. catenella, A. fundyense, A. minutum, A. tamarense*	[40,42,43,46,48]
	*sxtJ*	399–441	Regulatory	-	-
	*sxtK*	162	Regulatory	-	-
	*sxtS*	723–798	Ring formation	*A. minutum, G. catenatum, P. bahamense*	[43,48]
	*sxtU*	774–777	Short-chain alcohol dehydrogenase	*A. catenella, A. fundyense, A. minutum, A. tamarense, P. bahamense*	[42,43,46,48]
	*sxtV*	1650–1677	Dioxygenase reductase	-	-
	*sxtW*	324–327	Ferredoxin	*A. catenella*	[46]
Tailoring genes	*sxtL*	1269–1296	Decarbamoylation	*A. tamarense*	[48]
	*sxtN*	825–906	Sulfotransferase	*A. catenella, A. tamarense*	[42,48]
	*sxtO*	495–600	PAPS biosynthesis	*A. catenella*	[42,46]
	*sxtX*	753–771	*N*-1 hydroxylation	*A. catenella, A. tamarense, P. bahamense*	[42,46,48]
Regulator genes	*sxtY*	663	Signal transduction	-	-
	*sxtZ*	1350	Signal transduction	*A. catenella*	[42,46]
Transporter genes	*sxtF/M*	1413–1455	Export of PSTs	*A. catenella, A. fundyense, A. minutum, A. tamarense*	[42,43,46,48]
	*sxtP*	1125–1479	Binding of PSTs	*A. catenella, A. tamarense*	[46,48]
Unknown	*sxtE*	360–474	Unknown	-	-
	*sxtQ*	774	Unknown	-	-
	*sxtR*	744–879	Unknown	*A. fundyense, A. minutum*	[43]

**Table 3:** Summary of major findings on saxitoxin molecular biosynthesis in dinoflagellates using transcriptomics approach. EST—expressed sequence tag.

Studied Species	Experimental Design	Summary of Findings	Reference
*A. minutum*	Construction of EST library for *A. minutum*	In silico search against EST library failed to identify any homologues of cyanobacteria saxitoxin genes	[36]
*A. minutum*	Microarray-based analysis of differentially expressed nutrient and toxin-related genes	Two unannotated genes were expressed during toxin production	[62]
*A. fundyense* and *A. minutum*	Gene survey study using 454 sequencing	Two different *sxtA* transcripts were present in dinoflagellates transcriptome with the longer transcript (*sxtA4*) exclusive to saxitoxin-producing dinoflagellates, which are likely involved in saxitoxin biosynthesis	[43]
*A. tamarense, G. catenatum* and *P. bahamense*	Gene survey study using 454 sequencing and Illumina Hiseq	Several *sxt* genes present in the transcriptome; however, phylogenetic analysis of these genes indicated that saxitoxin biosynthesis in dinoflagellates and cyanobacteria is acquired independently	[48]
*A. minutum*	Microarray-based analysis of transcriptome response toward grazer-induced induction	Two unannotated genes showed consistent regulation pattern with saxitoxin content in dinoflagellates	[71]
*A. catenella*	Comparison of transcriptome profile obtained using Illumina Hiseq between toxic and non-toxic dinoflagellates	Long isoform of *sxtA* was highly downregulated in the non-toxic strain; s*xtO* and *sxtZ* were discovered in dinoflagellates for the first time	[42]
*Alexandrium* spp.	Screening and analysis of EST library for 36 dinoflagellate species for occurrence of *sxtA1*, *sxtA4*, and *sxtG* genes	*SxtA4* gene is highly conserved and exclusive to saxitoxin-producing dinoflagellates	[54]
*A. fundyense*	Metatranscriptome profiling during *A. fundyense* bloom in Northport/Huntington Bay complex, Long Island	*SxtA* expression was upregulated in the presence of low dissolved inorganic nitrogen in the environment	[41]
*A. minutum*	Transcriptome profiling and gene expression studies of several toxin-related genes under different nutritional conditions	*SxtA4*, *sxtI*, and *sxtG* gene expression patterns were consistent with toxin production	[40]
*S. trochoidea*	Transcriptome profiling under nitrate depletion	A total of 113 transcripts were recognized as homologues for *sxt* genes despite the fact that no saxitoxin is produced by this species; low transcriptional changes during nitrogen depletion detected	[60]
*A. catenella*	Transcriptome profiling at different toxin biosynthesis stages within cell cycle	138 homologues of *sxt* genes were identified; however, their expression patterns were inconsistent with toxin level, suggesting that saxitoxin regulation occurs at the post-transcriptional level	[46]

**Table 4:** Reports on translational control in dinoflagellates.

Findings	Reference
Enzyme in the TCA cycle exhibited circadian changes in accordance with protein abundance, whereas its messenger RNA (mRNA) level remained constant throughout the cycle	[84]
Presence of unique splice leader at 5’ of dinoflagellates mRNA might provide translational regulation in dinoflagellates via trans-splicing	[72,73]
Expression of conserved *S*-phase genes in *Karenia brevis* remains unchanged throughout cell cycle, but other protein expression level was observed	[85]
Presence of dinoflagellate spliced leader sequence at 5’ of *sxtA* and *sxtG* genes might indicate that saxitoxin biosynthesis is regulated at the translational level	[43,44]
Daily circadian system in dinoflagellate *Lingulodinium* showed lack of regulation at the transcript level using RNA-sequencing approach, suggesting the involvement of translational or post-translational control of this system	[86]
Identification of microRNAs (miRNAs) in several species of dinoflagellates, including saxitoxin-producing dinoflagellates, indicates regulation of several genes in dinoflagellates at post-transcriptional level via a small RNA gene silencing mechanism	[76,77,78,79,80]
Characterization of extensive transcript encoding protein elF4E family in dinoflagellates	[83]
Genome sequence of *Symbiodinium kawagutii* revealed substantial translational control by miRNA in biological processes involving carbohydrate metabolism, transcription regulation, and biosynthesis of amino acids and antibiotics	[79]
Poor correlation between protein and mRNA level in dinoflagellate *Lingulodinium*	[87]
